# Elevated expression of LAG-3, but not PD-1, is associated with impaired iNKT cytokine production during chronic HIV-1 infection and treatment

**DOI:** 10.1186/s12977-015-0142-z

**Published:** 2015-02-13

**Authors:** Jennifer A Juno, Andrew T Stalker, Jillian LM Waruk, Julius Oyugi, Makobu Kimani, Francis A Plummer, Joshua Kimani, Keith R Fowke

**Affiliations:** Department of Medical Microbiology, University of Manitoba, 539 – 745 Bannatyne Avenue, Winnipeg, Manitoba R3E 0 J9 Canada; National HIV and Retrovirology Laboratory, JC Wilt Infectious Disease Research Centre, Winnipeg, Manitoba Canada; Department of Medical Microbiology, University of Nairobi, Nairobi, Kenya; Department of Community Health Sciences, University of Manitoba, S113 - 750 Bannatyne Avenue, Winnipeg, Manitoba R3E 0 W3 Canada

**Keywords:** Exhaustion, iNKT cells, LAG-3 protein human, HIV, CD223, Immune dysfunction

## Abstract

**Background:**

LAG-3 is a potent negative regulator of the immune response but its impact in HIV infection in poorly understood. Unlike exhaustion markers such as PD-1, Tim-3, 2B4 and CD160, LAG-3 is poorly expressed on bulk and antigen-specific T cells during chronic HIV infection and its expression on innate lymphocyte subsets is not well understood. The aim of this study was to assess LAG-3 expression and association with cellular dysfunction on T cells, NK cells and iNKT cells among a cohort of healthy and HIV-infected female sex workers in Nairobi, Kenya.

**Results:**

Ex vivo LAG-3 expression was measured by multiparametric flow cytometry, and plasma cytokine/chemokine concentrations measured by bead array. Although LAG-3 expression on bulk T cells was significantly increased among HIV-infected women, the proportion of cells expressing the marker was extremely low. In contrast, LAG-3 was more highly expressed on NK and iNKT cells and was not reduced among women treated with ART. To assess the functional impact of LAG-3 on iNKT cells, iNKT cytokine production was measured in response to lipid (αGalCer) and PMA/Io stimulation by both flow cytometry and cytokine bead array. iNKT cytokine production is profoundly altered by both HIV infection and treatment, and LAG-3, but not PD-1, expression is associated with a reduction in iNKT IFNγ production.

**Conclusions:**

LAG-3 does not appear to mediate T cell exhaustion in this African population, but is instead expressed on innate lymphocyte subsets including iNKT cells. HIV infection alters iNKT cytokine production patterns and LAG-3 expression is uniquely associated with iNKT dysfunction. The continued expression of LAG-3 during treatment suggests it may contribute to the lack of innate immune reconstitution commonly observed during ART.

**Electronic supplementary material:**

The online version of this article (doi:10.1186/s12977-015-0142-z) contains supplementary material, which is available to authorized users.

## Background

During chronic HIV infection, persistent antigen exposure, microbial translocation and increasing immune activation lead to the phenomenon of immune exhaustion, where lymphocyte subsets (most commonly CD8+ T cells) exhibit a progressive loss of cytotoxicity, cytokine production and proliferation (reviewed in [1]). The phenotype of immune exhaustion is often mediated by the increased and simultaneous expression of inhibitory surface proteins such as PD-1, Tim-3, LAG-3, CD160 and 2B4/CD244 on CD4+ and CD8+ T cells. Initiation of combination antiretroviral therapy (cART) often returns inhibitory receptor levels to baseline [2,3], and blockade of these pathways during infection is being considered as a component of viral eradication strategies [4].

In contrast to highly studied markers such as PD-1 and Tim-3, less is known about the expression and role of LAG-3 in immune exhaustion during chronic HIV infection. The LAG-3 protein bears structural similarities to CD4 and binds the same ligand, MHC class II, but with much greater affinity [5-7]. LAG-3 cross-linking inhibits Ca^2+^ fluxes, T cell proliferation and IFNγ, TNFα and IL-2 (but not Th2 cytokine) production following TCR re-stimulation [8,9]. In healthy individuals, its *ex vivo* expression is negligible, but it is known to be expressed on activated T and NK cells [5,10], Tregs [11,12], iNKT cells [13] and some pDC populations [14]. Microarray studies of gene expression during multiple stages of HIV/SIV infection indicate modulation of LAG-3 expression [15,16]; LAG-3 transcript levels positively correlate with viral load set point and are significantly higher among rapid progressors compared to viremic non-progressors [17]. *In vitro,* HIV-pulsed DCs can induce the expression of a number of exhaustion markers, including LAG-3, on T cells [18].

Despite these observations, evidence of LAG-3 involvement in lymphocyte dysfunction during chronic infection remains elusive. With the exception of one report [19], early studies suggested that LAG-3 is not upregulated on bulk T cells during HIV infection [20]. Recent characterization of HIV-specific T cells found that the major populations of exhausted HIV-specific CD8+ and CD4+ T cells lack LAG-3 expression [21,22]. Although a recent study reported on a population of LAG-3+ HIV-specific CD8+ T cells associated with low viral loads, the authors were unable to detect LAG-3 expression on HIV-specific T cells prior to a 30 hour stimulation *in vitro* [23].

Importantly, LAG-3 expression on non-T lymphocyte subsets during chronic HIV infection has not been assessed. In HIV infection, NK cells exhibit multiple functional defects [24,25] that persist during cART [26], as well as expansion of a dysfunctional CD56^−^CD16^+^ subset that expresses high levels of inhibitory receptors [27]. The invariant NKT (iNKT) population is also affected by HIV infection. iNKTs are innate lymphocytes and a small subset of CD1d-restricted CD3+ lymphocytes that respond to lipid antigen stimulation with robust cytokine production and proliferation, creating a potent link between the innate and adaptive arms of the immune system. CD4+ iNKTs are depleted during chronic HIV infection [28,29] and exhibit variable reconstitution during ART [28,30,31]. The bulk iNKT population displays also defects in IFNγ/TNFα secretion and proliferation [30,32-34]. The consequences of iNKT dysfunction or exhaustion during infection could be considerable due to the unique role of iNKT cells in linking, and directing, innate and adaptive immunity. A hallmark of iNKT activation is the rapid production of a vast array of cytokines and chemokines [35-37]. Other iNKT effector functions include perforin/granzyme release associated with NKG2D engagement [38-42], and Fas/FasL-mediated cytotoxicity [36,39]. In addition to direct cytolytic function and cytokine secretion, iNKTs play an important role in the activation and regulation of other immune cell subsets. iNKT activation reciprocally modulates DC function [43-45] and quickly leads to NK cell activation in an IFNγ-dependent manner, followed by T and B cell activation [46]. Stimulation of iNKT cells in conjunction with soluble T cell antigen enhances both CD4+ and CD8+ antigen-specific responses [47,48].

Consequently, loss of iNKT function can lead to significant defects in adaptive immunity. Data from non-human primate studies suggests that pre-infection iNKT frequency correlates with retention of CD4 count following SIV challenge, and supernatant from iNKT cultures is sufficient to inhibit HIV replication *in vitro*. To date, the determinants of iNKT dysfunction during HIV infection are poorly understood. In one study, the exhaustion marker PD-1, which contributes to loss of T cell function in HIV infection, was unrelated to iNKT dysfunction [30], but the role of other inhibitory receptors such as LAG-3 has not been assessed.

In this context, several gaps in knowledge remain. Although T cell LAG-3 expression is generally low, no studies to date have included cohorts of African populations where T cell immune activation is significantly higher compared to Caucasians [49] and HIV clade A/D/C infections predominate. We also extended our studies of LAG-3 expression beyond CD4+ and CD8+ T cells, given reports of LAG-3 expression on activated NK and *ex vivo* iNKT cells and simultaneously assessed LAG-3 expression on T cell, NK and iNKT subsets among a population of healthy and HIV-infected Kenyan commercial sex workers. In a follow-up study, we determined the functional consequences of the association between LAG-3 expression and iNKT cytokine production in chronically infected and ART-treated individuals. This is the first study to functionally assess LAG-3 on multiple lymphocyte populations among an African cohort.

## Results

### LAG-3 Expression screening study

A total of 10 HIV-, 40 HIV+ ART naïve and 40 HIV+ ART experienced women were recruited for the study (Table 1). The gating strategy for *ex vivo* samples is shown in an (Additional file 1). CD3+ and CD3- subsets were identified by histogram gating. iNKT cells were identified as CD3 + 6B11+, while conventional T cells were identified as CD3 + 6B11- and further subgated based on CD4 and CD8 expression. NK cells were gated as CD56^hi^CD16-, CD56^dim^CD16+ or CD56-CD16+ within the CD3- population.

**Table 1:** **Characteristics of cohort subjects included in the screening study**

	**HIV-N**	**HIV+ ART naïve**	**HIV+ ART experienced**	**p value** ^**^
Age, years^*^	31.5 (28.75, 41)	37 (32.5, 41)	40 (34, 45.25)	0.164
Duration of sex work, years^*^	9 (5.25, 13.5)	7 (5, 14)	11.5 (5, 19)	0.552
CD4 Count^*^	--	428 (369.3, 632.3)	354 (245, 481)	**0.011**
Duration of ART, years^*^	--	--	2.14 (1.16, 3.64)	--

^*^Data are presented as median (IQR).

^**^Groups were compared by Kruskal-Wallis test (age, duration of sex work) or Mann–Whitney test (CD4 count).

**Figure 1:**
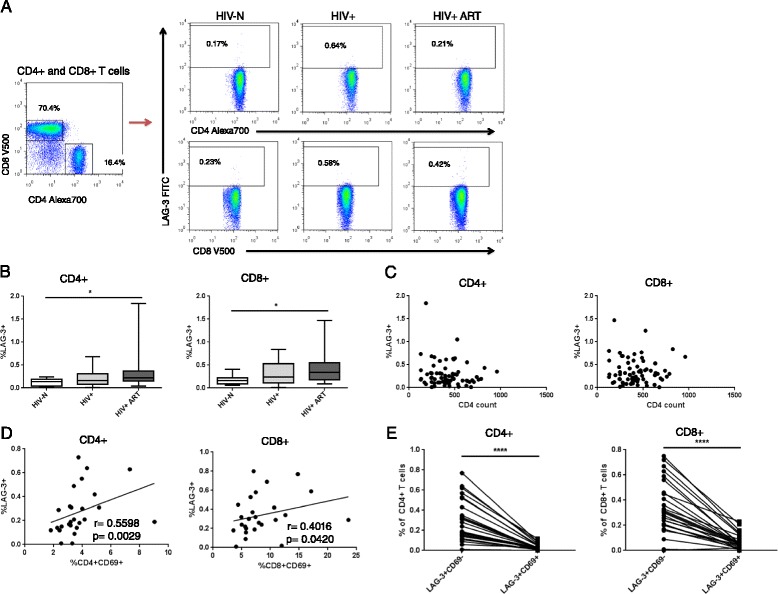
***Ex vivo*** **LAG-3 expression on CD4+ and CD8+ T cell subsets. (A)** Representative *ex vivo* surface staining of LAG-3 on CD4+ and CD8+ T cells. **(B)** LAG-3 expression on both CD4+ and CD8+ T cells among HIV-N, HIV+ and HIV+ ART groups. **(C)** Correlation between LAG-3 expression and CD4 count among HIV+ participants. **(D)** Correlation and **(E)** co-expression of LAG-3 and CD69 among all HIV+ participants. Statistical comparisons made by Kruskal-Wallis (with Dunn’s post-test), Wilcoxon test and Spearman correlation. *p < 0.05, ****p < 0.0001.

We first assessed LAG-3 expression on bulk CD4+ and CD8+ T cell subsets (Figure 1A). Consistent with reports in Caucasian populations [21], LAG-3 expression was negligible among healthy participants (median 0.14% CD4 + LAG-3+ and 0.165% CD8 + LAG-3+) (Figure 1B). LAG-3 expression was significantly affected by HIV infection on both subsets (p = 0.017 for CD4+, 0.018 for CD8+), with significant post-test differences between the HIV-N and HIV+ ART groups (p < 0.05 for CD4+ and CD8+) (Figure 1B). Despite the significant differences, LAG-3 expression remained extremely low among all three participant groups (<2% of CD4+ or CD8+ population).

Exhaustion marker expression typically correlates with disease progression, whether measured by viral load, CD4 count or immune activation [50,51]. Because viral load determination is not performed as the standard of care in Kenya and was not available for these participants, CD4 count and CD8+ T cell activation (measured by HLA DR expression) were used as surrogates of disease progression. LAG-3 expression did not correlate with CD4 count on either CD4+ or CD8+ T cells among either group of HIV+ participants (p > 0.1 for all HIV+, HIV+ ART naïve and HIV+ ART experienced) (Figure 1C). Similarly, LAG-3 expression did not correlate with CD8+ HLA DR expression on any cell subset among any HIV+ groups (not shown). In addition to CD8+ T cell activation being an indicator of disease progression, LAG-3 is reported to be expressed primarily on activated T cells. Therefore, we also assessed the correlation between LAG-3 expression and acute CD4+ and CD8+ T cell activation measured by CD69 expression. Notably, LAG-3 and CD69 expression significantly correlated on both CD4+ and CD8+ T cells (r = 0.560, p = 0.003 for CD4+; r = 0.402, p = 0.042 for CD8+) (Figure 1D).

To confirm that the negligible T cell LAG-3 expression in this cohort was specific to LAG-3 and not due to aberrant expression of all exhaustion markers, we jointly assessed LAG-3 and PD-1 expression in a small follow-up study of 15 HIV-N, 8 HIV+ and 17 HIV+ ART participants.

Consistent with all published literature [21], PD-1 expression was robust among all participants and was increased on CD8+ T cells among HIV-infected participants compared to healthy controls (p = 0.0425) (Additional file 2A). LAG-3 expression did not correlate with PD-1 expression on CD8+ T cells (Additional file 2B). CD8 + LAG-3+ T cells, however, expressed significantly more PD-1 than the bulk CD8+ T cell population (p < 0.0001) (Additional file 2C), indicating that LAG-3 tends to be co-expressed with PD-1 on CD8+ T cells during HIV infection.

**Figure 2:**
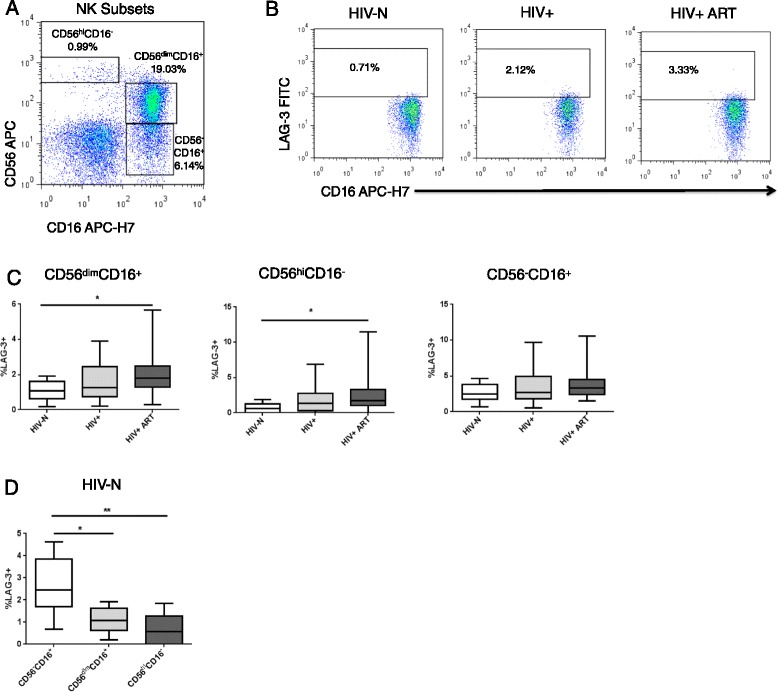
***Ex vivo*** **LAG-3 expression on NK cell subsets. (A)** Representative *ex vivo* surface staining of NK cell subpopulations and **(B)** LAG-3 expression on CD56^dim^CD16^+^ NK cells**. (C)** LAG-3 expression on the CD56dimCD16+ and CD56hiCD16- NK subsets among healthy and HIV-infected groups. **(D)** Paired analysis of LAG-3 expression across NK subsets among HIV-N participants. Statistical comparisons made by Kruskal-Wallis (with Dunn’s or Mann–Whitney post-test) or Friedman test. *p < 0.05, **p < 0.01.

We next quantified LAG-3 expression on multiple NK cell subsets based on CD56 and CD16 expression. As shown in Figure 2A, NKs were defined as CD56^hi^CD16^−^ (immunoregulatory), CD56^dim^CD16^+^ (cytotoxic) and CD56^−^CD16^+^ (dysfunctional/anergic) (based on [27,52]). While Tim-3 is reportedly expressed at high levels, particularly on CD56^dim^ NKs [53], LAG-3 expression should typically be restricted to activated NK cells. LAG-3 expression was detected at low levels on all NK subsets among healthy individuals (Figure 2A, B). HIV infection altered LAG-3 expression on both the CD56^dim^CD16^+^ and CD56^hi^CD16^−^ NK subsets, but not CD56^−^CD16^+^ NKs (CD56^dim^CD16^+^ p = 0.0368, CD56^hi^CD16^−^ p = 0.0134, CD56^−^CD16^+^ p = 0.1474) (Figure 2C). As observed with T cell LAG-3 expression, the significant differences were between the HIV+ ART experienced women compared to healthy controls (CD56^dim^CD16^+^ p = 0.0151, CD56^hi^CD16^−^ p < 0.05).

The relationship between NK LAG-3 expression and CD4 count, ART duration and CD4 recovery was virtually identical to that described for T cell subsets, and exhibited no significant associations. Intra-participant comparison of LAG-3 expression across NK subsets revealed significantly higher LAG-3 expression on the CD56^−^CD16^+^ NK subset compared to either the CD56^dim^ or CD56^hi^ populations. The differences remained significant when analysis was restricted to only HIV- individuals (p = 0.0004, post-test p < 0.05 for CD56- versus CD56dim, p < 0.01 for CD56- versus CD56hi) (Figure 2D) or when restricted to all HIV+ and HIV+ ART participants (p < 0.0001, not shown). A parametric ANOVA test for linear trend demonstrated a significant decline in LAG-3 expression across NK cell subsets with increasing CD56 expression (CD56- > CD56dim > CD56hi) (p < 0.0001, ANOVA test for linear trend).

**Figure 3:**
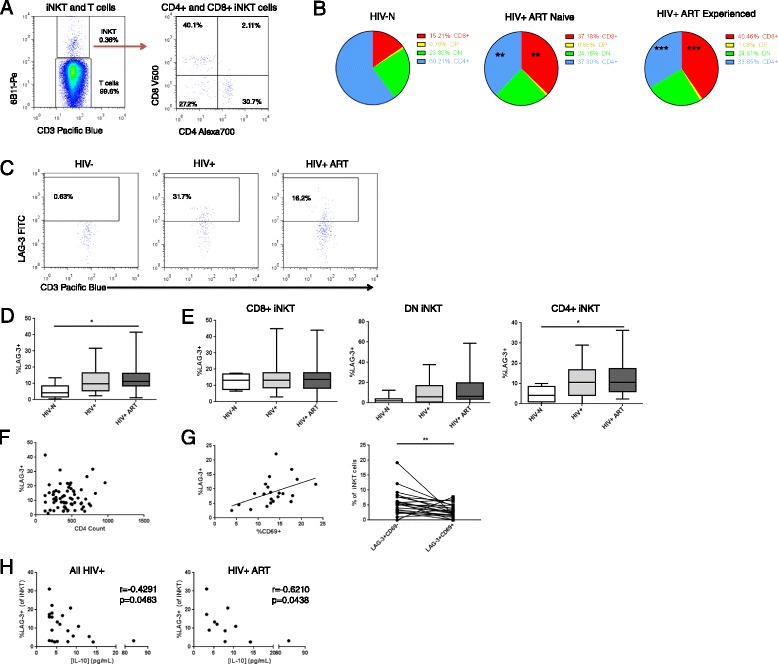
***Ex vivo*** **LAG-3 expression on iNKT cell populations. (A)** Representative *ex vivo* surface staining of iNKT cells (CD3 + 6B11+) and sub-gating into CD4+, CD8+ and double negative (DN) subsets. **(B)** Relative proportion of CD4+ iNKTs within the bulk iNKT population. **(C)** Representative staining of LAG-3 on CD3 + 6B11+ iNKT cells. **(D)** LAG-3 expression on bulk, as well as **(E)** CD8+, DN and CD4+ iNKT populations among study groups. **(F)** Correlation between LAG-3 expression and CD4 count among HIV+ participants. **(G)** Correlation and **(F)** co-expression between CD69 and LAG-3 on iNKT cells among HIV+ participants. **(H)** Correlation between iNKT LAG-3 expression and plasma IL-10 concentration among all HIV+ and HIV+ ART participants. Statistical comparisons made by Kruskal-Wallis (with Dunn’s post-test), Wilcoxon test and Spearman correlation *p < 0.05, **p < 0.01, ***p < 0.001.

Human iNKT population frequencies are highly variable, ranging from <0.01% of CD3+ T cells to >1% in published reports [13,54]. We used an antibody specific to the iNKT TCR (6B11) that is routinely used for iNKT identification [55-62] and that provides results comparable to those obtained using CD1d tetramers [63] (Figure 3A). Unlike murine iNKTs, human iNKT cells can be subgrouped as CD4+, CD8α + or double negative (DN) [13]. Some samples contained too few iNKT events for further analysis, thereby reducing sample sizes for this analysis to 8 HIV-N, 34 HV+, and 34 HIV+ ART participants. Similar to the depletion of conventional CD4+ T cells during chronic HIV infection, CD4+ iNKTs are easily infected and depleted [28]. Consistent with previous reports, the proportion of CD4+ iNKT cells in this cohort was reduced in the HIV+ ART naïve group (p = 0.0012, post-test p < 0.01 for healthy vs. HIV+). Interestingly, CD4+ iNKT proportion remained low among HIV+ ART experienced participants (p < 0.001 for healthy vs. HIV+ ART experienced) (Figure 3B), suggesting no reconstitution of the CD4+ iNKT compartment in this population during ART. The proportionate decrease in CD4+ iNKT cells was associated with an inflated proportion of CD8+ iNKT cells (p = 0.0005, post-test p < 0.01 for healthy vs. HIV+, p < 0.001 for healthy vs. HIV+ ART experienced), while the frequency of DN iNKTs remained similar across groups (Figure 3B).

LAG-3 expression was detected at low levels on iNKTs among healthy women, and was significantly elevated during chronic HIV infection among ART experienced participants (Figure 3C, D) (p = 0.0274, post-test p < 0.05 for HIV- versus HIV+ ART). LAG-3 expression on the bulk iNKT subset did not correlate with CD4 count among HIV+ participants, whether grouped together or stratified by ART use (Figure 3F) (p > 0.1 for all). iNKT CD4+/CD8+/double negative subset analysis of participants with sufficient events in each gate demonstrated that LAG-3 expression was significantly increased only on the CD4+ iNKT subset among HIV+ ART experienced individuals (p = 0.029, post-test p < 0.05 HIV- versus HIV+ ART) (Figure 3E). Among all HIV+ participants, iNKT LAG-3 expression did significantly correlate with iNKT activation as measured by CD69 expression (Figure 3G) (r = 0.4507, p = 0.0353).

Given that LAG-3 expression was largely unrelated to HIV disease progression, we assessed whether LAG-3 expression on any lymphocyte subset was related to plasma concentrations of IFNα2, IFNγ, IL-4, IL-6, IL-10, MIP-1β, sCD40L and TNFα quantified by cytokine bead array. Among participants with detectible analyte levels, iNKT LAG-3 expression exhibited a weak negative correlation with plasma IL-10 concentrations among HIV-infected participants as well as only ART experienced participants (r = −0.4291, p = 0.0463 for HIV+/HIV+ ART; r = −0.6210, p = 0.0438 for HIV+ ART) (Figure 3H).

### Assessment of iNKT function

Because LAG-3 was expressed at higher levels on iNKT cells than T cells or NK cells during HIV infection, we further explored the relationship between LAG-3 expression and iNKT cell function in HIV+ participants. A total of 16 HIV-uninfected, 9 HIV+ ART naïve, and 17 HIV+ ART experienced women were recruited for this follow-up study. The characteristics of these groups, including age, duration of sex work, CD4 count and duration of ART are described in Table 2.

**Table 2:** **Characteristics of follow-up study participants**

**Variable** ^*^	**HIV-negative**	**HIV+ ART naïve**	**HIV+ ART experienced**	**p value** ^**^
Age, years	40 (34, 44.5)	35 (29, 41)	40 (34.5, 44)	0.522
Duration of sex work	10 (6.5, 12)	9 (5, 14)	11 (8, 20)	0.324
CD4 Count	--	475.5 (321, 618)	526.5 (407, 698)	0.297
Duration of ART	--	--	3 (3, 5.5)	--

^*^Data are presented as median (IQR).

^**^Groups were compared by Kruskal-Wallis test (age, duration of sex work) or Mann–Whitney test (CD4 count).

**Figure 4:**
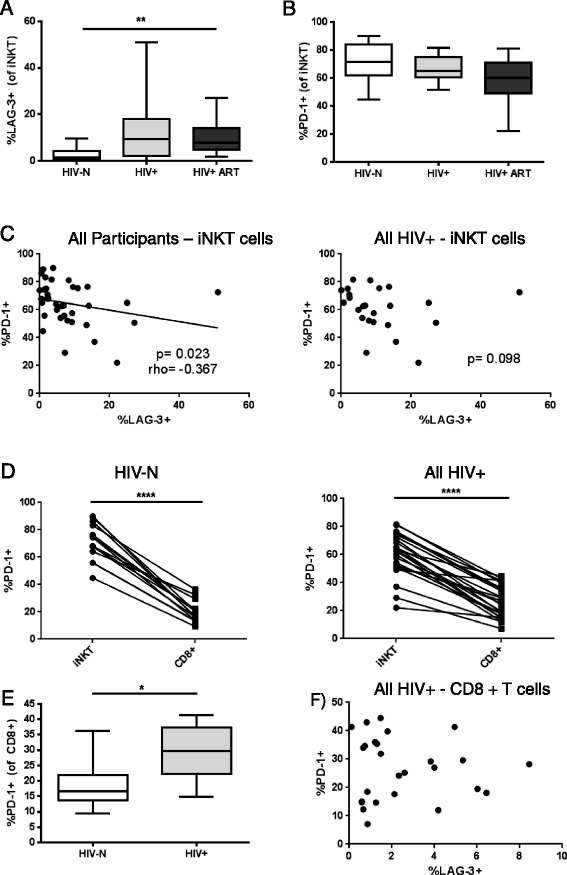
**Baseline iNKT LAG-3 and PD-1 expression among healthy HIV-N (n = 14), HIV+ (n = 9) and HIV+ ART (n = 15) women.** Comparison of **(A)** iNKT LAG-3 expression and **(B)** iNKT PD-1 expression among HIV-N, HIV+ and HIV+ ART groups in a follow-up study. **(C)** Correlation between LAG-3 and PD-1 expression all participants and HIV+/HIV+ ART women. **(D)** Comparison of PD-1 expression on iNKTs compared to conventional CD8+ T cells among both healthy and HIV-infected women. **(E)** PD-1 expression on CD8+ T cells among HIV+ ART naïve women (n = 9) compared to healthy controls (n = 16). **(F)** Correlation between LAG-3 and PD-1 on CD8+ T cells. Statistical comparisons made by Kruskal-Wallis (with Dunn’s post-test), Wilcoxon test, Mann–Whitney and Spearman correlation *p < 0.05, **p < 0.01, ****p < 0.0001.

Consistent with the screening study, LAG-3 expression was elevated on iNKT cells during HIV infection (p = 0.004), and was not restored to baseline by ART, as post-tests revealed significant differences between the HIV-N and HIV+ ART experienced groups (p < 0.01) (Figure 4A). In contrast to LAG-3, iNKT PD-1 expression was similar across groups (Figure 4B). Among all participants, iNKT LAG-3 expression weakly but inversely correlated with PD-1 expression (p = 0.023, r = −0.367), a relationship that persisted as a trend among HIV-infected (HIV+ and HIV+ ART) women (p = 0.098) (Figure 4C). Interestingly, the relationship between PD-1 and LAG-3 expression, and even PD-1 expression itself, on iNKT cells was distinct from the conventional CD8+ T cell subset. PD-1 expression on the iNKT subset was significantly higher than on CD8+ T cells among all participants (p < 0.0001) or when stratified by HIV status (p < 0.0001 for both HIV-N and combined HIV+ and HIV+ ART) (Figure 4D). Unlike the iNKT subset, PD-1 expression was significantly elevated on CD8+ T cells among HIV+ ART naïve participants compared to healthy controls (p = 0.010) (Figure 4E) and no correlation between CD8+ T cell LAG-3 and PD-1 expression was observed among HIV+ participants (p = 0.897) (Figure 4F).

### Assessment of iNKT cytokine production

**Figure 5:**
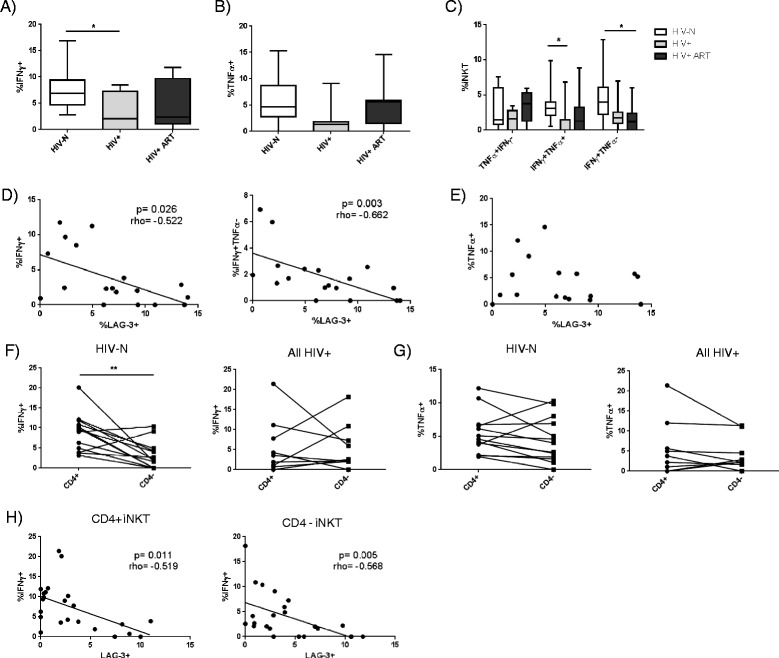
**Functional iNKT cytokine responses to αGalCer stimulation among healthy HIV-N (n = 15), HIV+ (n = 7) and HIV+ ART women (n = 11).** PBMC were stimulated for 10 hours with 100 ng/mL αGalCer, and iNKT production of **(A)** IFNγ and **(B)** TNFα were compared between study groups. **(C)** Analysis of mono- and dual-functional iNKT αGalCer responses between study groups. Correlation between iNKT LAG-3 expression and **(D)** IFNγ or **(E)** TNFα expression. Comparison of **(F)** IFNγ and **(G)** TNFα production by CD4+ and CD4- iNKTs among HIV-N and HIV+/HIV+ ART participants. **(H)** Correlation between LAG-3 and IFNγ expression among CD4+ and CD4- iNKT subsets. Statistical comparisons made by Kruskal-Wallis (with Dunn’s post-test), Wilcoxon test, and Spearman correlation. *p < 0.05, **p < 0.01.

To assess iNKT cytokine production in the Majengo cohort, PBMC from HIV-, HIV+ and HIV+ ART experienced participants were stimulated with either the iNKT lipid antigen αGalCer or the mitogen PMA/Io. IFNγ and TNFα were measured by intracellular flow cytometry (Additional file 3). Following stimulation with αGalCer, HIV status significantly affected IFNγ production by iNKT cells (p = 0.019) (Figure 5A), with HIV+ ART naïve participants producing significantly less IFNγ compared to healthy controls (post-test p < 0.05). IFNγ production was only partially restored among the HIV+ ART group, as the median%IFNγ + cells remained lower than that of healthy controls (2.43% for HIV+ ART compared to 6.89% for HIV-N and 2.06% for HIV+). TNFα production was more similar across subject groups, with only a trend toward differences in cytokine production based on HIV status (p = 0.058) (Figure 5B). Similar to the results observed for IFNγ, the HIV+ ART naïve group exhibited the lowest median cytokine production (1.29% TNFα + for HIV+ ART naïve compared to 4.74% for HIV-N and 5.62% for HIV+ ART). When analysing mono- versus dual-functional iNKT cells, HIV+ ART naïve participants responded to stimulation with significantly fewer double positive TNFα + IFNγ + cells than healthy controls (p = 0.030, post-test p < 0.05) (Figure 5C), while the HIV+ ART group exhibited significantly fewer TNFα-IFNγ + cells in response to lipid stimulation compared to uninfected women (p = 0.017, post-test p < 0.05) (Figure 5C).

Both bulk IFNγ production and single-positive TNFα-IFNγ + cell frequencies inversely correlated with iNKT LAG-3 expression among HIV+ and HIV+ ART women (p = 0.026, r = −0.522, and p = 0.003, r = −0.662, respectively) (Figure 5D). In contrast, LAG-3 expression did not correlate with bulk TNFα (Figure 5E), TNFα + IFNγ- or TNFα + IFNγ + responses (not shown). *Ex vivo* PD-1 expression did not significantly correlate with either bulk IFNγ or TNFα cytokine production following stimulation (not shown).

When comparing cytokine expression between CD4+ and CD4- iNKT subsets, HIV-N individuals exhibited significantly higher IFNγ production by CD4+ iNKTs (p = 0.006, Figure 5F), a difference which was lost among HIV+ and HIV+ ART participants (Figure 5F). In contrast, TNFα production was equivalent between iNKT subsets in both the HIV-N and HIV+/HIV+ ART groups (Figure 5F). Despite subset-specific differences in IFNγ production, *ex vivo* subset-specific LAG-3 expression inversely correlated with IFNγ production for both the CD4+ (p = 0.011, r = −0.519) and CD4- (p = 0.005, r = −0.568) subsets (Figure 5G). The correlation remained significant even after restricting analysis to HIV+ and HIV+ ART participants only (p = 0.040, r = −0.663 for CD4+ and p = 0.012, r = −0.735 for CD4-, not shown).

**Figure 6:**
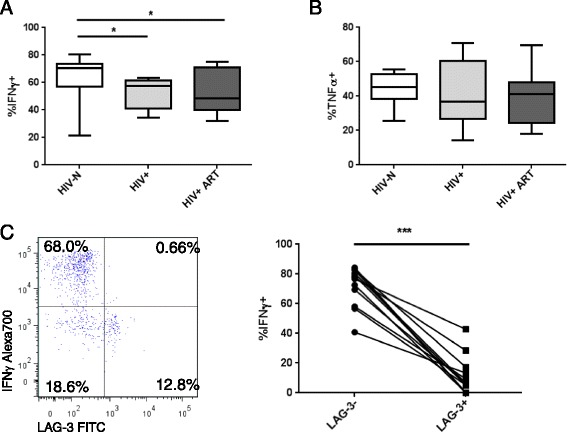
**PMA/Io-induced iNKT functional responses.** PBMC were stimulated for 6 hours with 25 ng/mL PMA and 500 ng/mL Io, and iNKT **(A)** IFNγ production and **(B)** TNFα production were compared among study groups. **(C)** Comparison of IFNγ production by LAG-3+ and LAG-3- iNKTs among all participants. Statistical comparisons made by Kruskal-Wallis (with Mann–Whitney post-test), Wilcoxon test and Spearman correlation. *p < 0.05, ***p < 0.001.

PMA/Io stimulation induced stronger cytokine responses compared to αGalCer among all participant groups. Interestingly, IFNγ secretion in response to PMA/Io stimulation varied significantly among groups (p = 0.034) (Figure 6A). Although no significant intra-group differences were detected by Dunn’s post-test, Mann Whitney comparison of each group demonstrated significantly lower IFNγ production among both the HIV+ ART naïve and HIV+ ART groups compared to healthy controls (p = 0.019 and 0.041, respectively). In contrast, PMA/Io-induced TNFα secretion was similar among all groups (p = 0.334) (Figure 6B). The high levels of cytokine and LAG-3 expression induced by PMA/Io stimulation allows for a comparison of IFNγ secretion by LAG-3+ and LAG-3- iNKT cells. Among all participants with sufficient iNKT cells for sub-gating, LAG-3+ iNKTs were significantly less likely to be IFNγ + than LAG-3- iNKTs (p = 0.0005, Figure 6C), confirming an inhibitory activity of LAG-3 on IFNγ production by iNKT cells.

### 5 day αGalCer-stimulated cytokine and sLAG-3 production

To investigate the downstream consequences of iNKT dysfunction during HIV infection of PBMC cytokine/chemokine production, we collected cell culture supernatants from 5 day iNKT αGalCer stimulations and quantified the expression of IFNγ, IL-4, IL-10, IL-13, IL-17, IP-10, MIP-1α, MIP-1β, and TNFα. IL-17 was detected above background by <50% of participants in each group, and was therefore excluded from further analysis.

**Figure 7:**
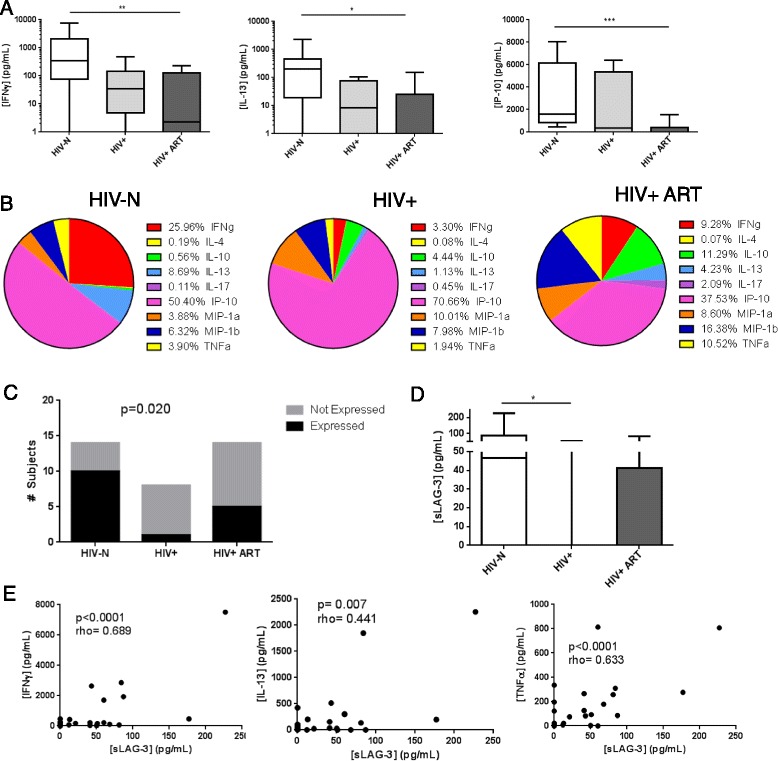
**Cytokine/chemokine and sLAG-3 responses 5 days post-αGalCer stimulation among HIV- (n = 15), HIV+ (n = 8) and HIV+ ART (n = 14) experienced participants.** PBMC were stimulated for 5 days with 100 ng/mL αGalCer, and cytokine/chemokine/sLAG-3 concentrations were quantified in the supernatant. **(A)** Supernatant concentrations of IFNγ, IL-13 and IP-10 were reduced among HIV-infected participants. **(B)** The proportional contribution of each analyte to total cytokine/chemokine expression among participant groups is shown. Aside from IP-10, HIV-N cytokine production is dominated by IFNγ and IL-13, while HIV+ cytokine production is dominated by TNFα and chemokine expression. **(C)** Proportion of subjects exhibiting above-background sLAG-3 production. **(D)** sLAG-3 concentration among study groups. **(E)** Correlation of sLAG-3 concentration IFNγ, IL-13 and TNFα among all participants. Statistical comparisons made by Kruskal-Wallis (with Dunn’s post-test), chi square test and Spearman correlation *p < 0.05, **p < 0.01, ***p < 0.001.

The proportion of individuals in each group demonstrating above-background expression of each analyte and the background subtracted concentration of each analyte are reported in Table 3. Significant differences in analyte concentrations across groups were detected for IFNγ, IL-13 and IP-10 (p = 0.003, 0.014 and 0.001, respectively, Figure 7A), and there was a trend toward a reduction in IL-4 concentration (p = 0.095). Post-tests revealed that inter-group differences were primarily significant for the HIV-N versus HIV+ ART experienced group comparisons (p < 0.01, <0.05, <0.001, respectively). Given that iNKT activation contributes to NK, B and T cell activation, these cell types most likely contributed to the cytokine/chemokine milieu by 5 days post-stimulation. *Ex vivo* iNKT frequency significantly correlated with background-subtracted expression of all analytes except IL-10 (not shown), suggesting, that cytokine production was indeed iNKT-driven. Following adjustment for baseline iNKT frequency, significant differences between groups in IFNγ (p = 0.009) and IP-10 (p = 0.006) expression remained, as well as a trend toward differences in IL-13 (p = 0.055) expression (not shown).

**Table 3:** **Above-background cytokine responses to αGalCer at 5 days post-stimulation**

**Analyte**		**HIV-negative (n = 14)**	**HIV+ ART naïve (n = 8)**	**HIV+ ART experienced (n = 14)**	**p value** ^**^	**Post-test differences** ^***^
IFNγ	N Detectible	13	7	8	**0.057**	
	Concentration^*^	**345.9 (76.74, 2115)**	**34.97 (4.83, 142.7)**	**2.275, (0.0, 124.8)**	**0.003**	**HIV-N vs HIV+ ART**
IL-4	N Detectible	7	3	2	0.129	
	Concentration^*^	3.06 (0.0, 22.02)	0.0 (0.0, 6.69)	0.0 (0.0, 0.18)	**0.095**	
IL-10	N Detectible	4	2	6	0.618	
	Concentration^*^	0.0 (0.0, 5.82)	0.0 (0.0, 4.25)	0.0 (0.0, 13.8)	0.683	
IL-13	N Detectible	11	5	6	0.152	
	Concentration^*^	**199.1 (19.23, 477.0)**	**8.26 (0.11, 77.2)**	**0.0 (0.0, 25.45)**	**0.014**	**HIV-N vs HIV+ ART**
IP-10	N Detectible	**11**	**6**	**5**	**0.005**	
	Concentration^*^	**1586 (836.8, 6168)**	**329.3 (24.5, 5351)**	**0.0 (0.0, 409.6)**	**0.001**	**HIV-N vs HIV+ ART**
MIP-1α	N Detectible	6	4	4	0.409	
	Concentration^*^	0.0 (0.0, 198.4)	37.2 (0.0, 158.5)	0.0 (0.0, 75.6)	0.453	
MIP-1β	N Detectible	8	4	4	0.368	
	Concentration^*^	78.16 (0.0, 370.2)	35.06 (0.0, 165.1)	0.0 (0.0, 104.6)	0.253	
TNFα	N Detectible	**10**	**7**	**6**	**0.084**	
	Concentration^*^	52.34 (0.0, 317.1)	20.2 (4.74, 115.1)	0.065 (0.0, 140.2)	0.370	

^*^Data are presented as median (IQR) in pg/mL.

^**^p values obtained from Chi squared tests for N detectible data, Kruskal-Wallis tests for concentration comparisons.

^***^Dunn’s post-test comparisons with p < 0.05 are indicated for each significant Kruskal-Wallis result Statistical tests with p ≤ 0.1 (trend) are indicated in bold.

In addition to differences in absolute levels of cytokine production, some studies have described cytokine production patterns by expressing the contribution of each analyte as a proportion of the total cytokine production by a group [64]. In this analysis, the concentration of each analyte was summed across all members of a group, and expressed as a percent of the total cytokines produced by that group (Figure 7B). In the HIV-N group, the αGalCer-induced cytokine response is dominated by IP-10, IFNγ and IL-13 expression. In contrast, the HIV+ ART naïve group demonstrated a relative expansion in the contribution of IP-10, MIP-1α and MIP-1β to the total cytokine environment. Although the relative proportions of IFNγ and IL-13 were somewhat restored in the ART experienced group, there was a strong expansion in the contribution of IL-10, MIP-1α, MIP-1β and TNFα to the total cytokine/chemokine environment that did not reflect either the HIV-N or HIV+ ART naïve groups.

We assessed whether e*x vivo* iNKT LAG-3 or PD-1 expression was related to cumulative αGalCer-induced cytokine/chemokine expression, although changes in LAG-3 and PD-1 expression over the course of cell culture and the contribution of non-iNKT cells to cytokine concentrations require that the data be interpreted with caution. Interestingly, iNKT LAG-3 expression continued to inversely correlate with IFNγ and IP-10 concentrations (r = −0.492, p = 0.004 and r = −0.458, p = 0.014, respectively), and trended toward an inverse correlation with IL-13 (p = 0.089) among all participants (Additional file 4). In contrast, PD-1 positively correlated with IFNγ, IL-4 and IP-10 concentrations among all participants (r = 0.399, p = 0.024; r = 0.512, p = 0.003; and r = 0.451, p = 0.016, respectively) (Additional file 4).

sLAG-3 concentration was quantified in the 5 day αGalCer stimulated cultures to determine whether large quantities of sLAG-3 were associated with a reduction in cytokine secretion. sLAG-3 production above background was detectible in 16/37 samples. Chi square analysis of the proportion of participants exhibiting above-background sLAG-3 levels showed significant differences between groups (p = 0.020), with HIV+ ART naïve patients being highly unlikely to produce sLAG-3 in response to stimulation (Figure 7C). sLAG-3 concentration varied significantly across groups (p = 0.017), with post-tests demonstrating significant differences between HIV-N and HIV+ ART naïve groups (p < 0.05) (Figure 7D). Following adjustment for *ex vivo* iNKT frequency, significant differences in sLAG-3 production remained (p = 0.026, HIV-N vs HIV+ post-test p < 0.05). Among all participants, sLAG-3 concentration significantly and positively correlated with all analytes except IL-10 and IL-4 (Additional file 5, Figure 7E). Among HIV-N participants, sLAG-3 significantly correlated with only IFNγ and TNFα (Additional file 5).

## Discussion

The major goals of this study were to assess T cell LAG-3 expression in a population with high immune activation and to compare LAG-3 expression levels on T cell, NK and iNKT cell populations during chronic infection and treatment. To our knowledge, this is the first report of T cell LAG-3 expression in an African cohort that is known to have high levels of immune activation. Although T cell LAG-3 expression was significantly elevated among HIV-infected participants compared to healthy controls, its expression remained extremely low among all individuals. These results are consistent with some reports of CD4 + LAG-3+ and CD8 + LAG-3+ T cell frequency during HIV infection [20], but not all [21]. It is interesting that the participants with the highest levels of LAG-3 expression were ART experienced, given that exhaustion and activation marker expression generally declines following ART initiation, even though immune function is not fully restored [2,3]. Although the cohort described by Yamamoto *et al.* [21] did demonstrate a decline of LAG-3 expression on bulk CD8+ T cells, they noted that LAG-3 expression on central memory cells (the memory subset with highest LAG-3 expression) did not decline among ART experienced patients, further suggesting that LAG-3 expression is not affected by viral suppression.

Surprisingly, the frequency of LAG-3+ cells in any lymphocyte subset studied was unrelated to disease progression (measured by CD4 count or CD8+ HLA DR expression) or ART duration. This is highly unusual compared to other exhaustion markers, which generally correlate well with multiple measures of disease progression and viremia [50,65,66]. Although LAG-3 expression correlated with CD69 expression, the two markers were rarely co-expressed. This likely reflects upregulation of LAG-3 following acute cellular activation, but with expression kinetics that are distinct from those of CD69.

The weak correlation between iNKT LAG-3 expression and plasma IL-10 concentration was the only correlation observed between lymphocyte LAG-3 expression and plasma cytokines or chemokines. It is, therefore, difficult to determine what biological marker drives LAG-3 expression. It is possible that LAG-3 expression on iNKT cells directly regulates IL-10 production, but perhaps more likely that the relationship with IL-10 reflects cross-talk between iNKT cells and other IL-10-producing lymphocyte subsets such as Tregs or neutrophils, both of which can interact with iNKTs [67,68]. Given that IL-10 can correlate with viral load [69], it is also possible that this relationship reflects a dependence of LAG-3 expression on disease progression, although that seems less likely given the lack of correlation between LAG-3 and CD4 count in this cohort.

Unlike Tim-3 [70], LAG-3 was not highly expressed on any NK cell subset. The differences in LAG-3 expression between NK subsets, however, warrant further investigation. Although LAG-3 expression was not elevated on CD56-CD16+ NK cells among HIV+ participants, its expression was highest on this subset among healthy controls. The expansion of this subset during HIV infection has been documented [71], and LAG-3 could contribute to the anergic phenotype observed among these cells.

In contrast to the T cell, and to some extent NK cell, subsets, LAG-3 was expressed at moderate levels on iNKT cells, particularly among the HIV-infected participants. In some patients, up to 40% of the iNKT population was LAG-3+, raising the possibility that iNKT cell function may be regulated by LAG-3 to a greater extent than other lymphocyte populations. iNKT cells express surface markers characteristic of both T and NK cells and respond to lipid antigen presentation by CD1d. Despite the low frequency of the iNKT population in the periphery (0.01 – 1% of CD3+ lymphocytes in humans), iNKT activity is now appreciated to play an important role in immunity to infectious diseases [72]. During chronic HIV infection, the CD4+ iNKT subset is depleted [28,29], as observed in our present cohort. While some cohorts report full recovery of both CD4+ and CD4- iNKT subsets following ART [31], others report no effect of therapy [28,30] similar to the lack of CD4+ iNKT reconstitution seen during this study.

Surprisingly, the increase in LAG-3 expression did not correspond to increased PD-1 expression, in contrast to another report by Moll *et al.* [30]. In both cohorts, iNKT PD-1 expression was several-fold higher than T cell PD-1 expression, but PD-1 was more readily detected on both cell subsets in our cohort. Differences in cohort ethnicity could contribute to the contrasting observations, but it is also plausible that the use of the EH12.2H7 anti-PD-1 clone coupled to the extremely bright Brilliant Violet 421 fluorochrome enabled more sensitive detection of PD-1 expression in this study than other clones and fluorochromes.

To determine the impact of iNKT LAG-3 expression, we assessed functional iNKT cytokine production capacity in the Majengo cohort. Studies of iNKT function during HIV infection and treatment in the literature suffer from lack of replication in multiple cohorts, and the only functional study performed in an African cohort reported only PMA/Io-stimulated cytokine production from ART-naïve subjects [33]. The decrease in αGalCer-induced IFNγ expression is similar to other cohorts, but the lack of functional reconstitution among ART experienced participants in this cohort is concerning [30,34]. Data on iNKT recovery during ART is inconsistent, as some studies suggest an improvement of iNKT cytokine production following ART [32,73], while other studies have failed to find ART-based improvement of iNKT function [30], or have not differentiated between treated and untreated subjects [34].

In this study, the *ex vivo* expression of LAG-3 on the iNKT subset inversely correlated with IFNγ secretion as measured by ICS following αGalCer stimulation. The consistent relationship between LAG-3 expression and IFNγ secretion for both CD4+ and CD4- iNKTs suggests a similar consequence of LAG-3 expression on both subsets, despite differences in IFNγ production between subsets in healthy individuals. The inhibitory activity of LAG-3 on iNKT IFNg expression was further confirmed by the demonstration that LAG-3+ iNKTs are highly unlikely to produce IFNγ following PMA/Io stimulation. The association of LAG-3, but not PD-1, with iNKT dysfunction during HIV infection is broadly consistent with a previous study of iNKT PD-1 expression [30]. Although PD-1 expression was elevated among HIV-infected subjects in that cohort, its expression did not correlate with cytokine production or proliferation, and blocking of the PD-1/PD-L1 pathway did not improve proliferation or IFNγ secretion. To date, the contribution of other exhaustion markers (Tim-3, CD160, 2B4) to iNKT function during HIV infection has not been defined. The contribution of Tim-3 to iNKT inhibition during herpes simplex virus infection was assessed, but found to be not responsible for the defects in cytokine secretion [74].

The only surface marker previously demonstrated to inversely correlate with iNKT cytokine production during HIV infection is the costimulatory molecule CD161 [34]. CD161 is expressed by a variable, but generally high, proportion of iNKT cells [34,75,76]. The regulatory nature of CD161 on iNKT cells has not been fully clarified, as CD161+ iNKT cells are more likely to produce IFNγ and TNFα than CD161- cells in healthy individuals [76,77]. Our study, however, did not assess CD161 expression and therefore cannot confirm or rule out a contribution of CD161 upregulation to iNKT dysfunction.

To complement the ICS data, we also assessed cytokine/chemokine expression in cell culture supernatant after 5 days of αGalCer stimulation. In this assay, the background subtracted supernatant protein levels represent both iNKT cytokine expression and protein expression by other cell subsets in response to iNKT activation. As such, this data represents the downstream, cumulative impact of lipid stimulation/iNKT activation in a mixed culture. Give that iNKT activation is known to regulate a large number of other cell types, and that the functional impact of LAG-3 expression (which binds MHCII proteins) can only be truly assessed when MHCII-expressing cells are present, measuring cytokines in a whole PBMC culture may be more relevant than sorting and isolating iNKT cells. The correlation between iNKT frequency and analyte concentration strongly suggests that the measured analyte levels are iNKT-induced.

Overall, several patterns emerged from analysis of cytokine/chemokine expression levels and correlations. Among HIV-N participants, IFNγ and IL-13 were the major cytokines produced in culture after IP-10. IFN-γ is a hallmark cytokine produced upon αGalCer stimulation, and IL-13 is produced by activated iNKT cells and directs monocyte to DC differentiation [78]. The loss of IL-13 secretion among HIV+ participants likely reflects, at least partially, the depletion of the CD4+ iNKT subset, which is reportedly the sole iNKT subset to produce IL-13 [64,79]. In contrast, cytokine production among HIV+ ART naïve participants was mostly limited to IP-10 and MIP-1α/β production. The ART experienced group never produced significantly higher levels of any analyte compared the ART naïve group, and showed little evidence of iNKT reconstitution. These data suggest that in addition to a lack of reconstitution of iNKT function among ART experienced patients, the cytokine production profile is altered in comparison to both HIV-N and ART-naïve subjects.

The correlations between sLAG-3 production and cytokine/chemokine concentrations among all study groups suggest that sLAG-3 may simply be a marker of iNKT activation. There is no evidence that increasing concentrations of sLAG-3 in culture results in inhibition of cytokine production. The lack of production of sLAG-3 in HIV+ ART naïve, but not ART-treated, participants is distinct compared to other cytokine/chemokine responses, which were generally absent in the ART treated group. The production of sLAG-3 by iNKT cells following stimulation has not been described in the literature, and represents a significant gap in knowledge.

The correlation between LAG-3 expression and supernatant IFNγ at 5 days post-stimulation is consistent with the ICS results described above. It is important to note, however, that changes in LAG-3 expression during culture, and the contribution of other PBMC cell subsets to cytokine production make a direct link between LAG-3 expression and long-term cytokine production difficult. The positive correlations between PD-1 expression and cytokine production also suggest that PD-1 may indicate a more activated, rather than exhausted, iNKT phenotype.

In conclusion, we have demonstrated both short-term and long-term defects in iNKT cytokine/chemokine expression in response to lipid stimulation in a Kenyan cohort of HIV+ ART naïve and experienced women. Although PD-1 expression did not appear to mediate iNKT dysfunction, the elevated expression of LAG-3 was associated with an inhibition of IFNγ production, suggesting that LAG-3 at least partially regulates iNKT function during chronic infection. Coupled with previous observations from our lab, and others, that LAG-3 is poorly expressed on T cells during HIV infection, blockade of LAG-3 signaling may selectively restore innate immune function and, therefore, improve current treatment regimens.

## Conclusions

Overall, our data suggest that LAG-3 is unlikely to play a significant role in T cell immune exhaustion in an African population. Unexpectedly, LAG-3 upregulation during chronic HIV infection and treatment may be restricted to the iNKT innate lymphocyte subset. The maintenance of LAG-3 expression during ART suggests that it may contribute to the innate immune dysregulation observed among ART subjects, as well as contributing to the loss of iNKT cytokine production observed during chronic infection. Future studies that demonstrate the cell-intrinsic activity of LAG-3 expression will determine whether blocking LAG-3 activity could improve innate immune responses initiated by iNKT cells during HIV infection or treatment.

## Methods

### Study subjects (n = 90)

This study recruited 10 HIV-uninfected, 40 HIV-infected antiretroviral naïve and 40 HIV-infected antiretroviral experienced patients from the Majengo commercial sex worker cohort located in Nairobi, Kenya. In a follow-up study, an additional 16 HIV-N, 9 HIV+ and 17 HIV+ ART participants were recruited. The Majengo cohort is an open cohort established in 1984 that recruits both HIV- and HIV+ commercial sex workers. During a routine clinic visit, participants provided peripheral blood samples, and CD4 count was determined for HIV+ participants. The study was approved by ethics boards at both the University of Manitoba and the University of Nairobi, and all participants provided informed consent.

### Flow cytometric analysis

Peripheral blood mononuclear cells (PBMCs) were isolated from whole blood by Ficoll gradient separation. After isolation, cells were immediately stained for *ex vivo* flow cytometric analysis and plasma samples were collected and frozen for cytokine bead array assay. The following fluorochrome-conjugated antibodies were used in various combinations: LAG-3 FITC (clone 17B4, Enzo Life sciences), iNKT TCR PE (clone 6B11), CD69 PeCy5, CD3 Pacific Blue, CD8 V500, CD56 APC, CD4 Alexa700, CD16 APC-H7, HLA DR APC-H7, CD3 Alexa700, CD3 PeCy5, TNFα PeCy7, IFNγ Alexa700 (all from BD Biosciences), and PD-1 Brilliant Violet 421 (clone EH12.2H7, Biolegend), Live Dead viability dye (Invitrogen). Depending on the experiment, 1 – 2×10^6^ cells were stained for surface markers for 30 minutes at 4°C. Cells were resuspended in 1% paraformaldehyde solution and acquired on the BD LSRII flow cytometer. A minimum of 100,000 lymphocyte events were collected using BD FACS Diva. Analysis was performed using FlowJo version 7.5 (Treestar).

For stimulation experiments, cells were either stimulated with 25 ng/mL PMA and 500 ng/mL ionomycin for 6 hours, or 100 ng/mL of αGalCer for 10 hours. Golgi stop and golgi plug were added to the cell culture 2 hours post-stimulation to capture cytokine production. Cells were surface stained above, permeabilized with BD Cytofix/Cytoperm solution and stained for IFNγ and TNFα for 30 minutes at 4°C. Because αGalCer stimulation was observed to strongly downregulate the iNKT TCR, the 6B11 antibody was added at the time of stimulation to identify cells with downregulated TCR, similar to protocols for CD8+ T cell tetramer staining. Cells were also stimulated with 100 ng/mL αGalCer for 5 days and the culture supernatant collected for quantification of cytokine secretion by bead array.

### Plasma cytokine and chemokine concentrations

Concentrations of cytokines and chemokines in plasma samples were quantified using Milliplex MAP bead array kits (Millipore) according the manufacturer’s 2 hour protocol. The Human Cytokine/Chemokine bead panel included the following analytes (with given limits of detection): IFNα2 (7.2 pg/mL), IFNγ (2.4 pg/mL), IL-4 (0.6 pg/mL), IL-6 (0.4 pg/mL), IL-10 (0.5 pg/mL), MIP-1β (4.8 pg/mL), sCD40L (9.9 pg/mL) and TNFα (1.6 pg/mL). Cell culture supernatants from the iNKT αGalCer stimulations were analysed for expression of the following analytes (with corresponding sensitivities): IFNγ (0.8 pg/mL), IL-4 (4.5 pg/mL), IL-10 (1.1 pg/mL), IL-13 (1.3 pg/mL), IL-17 (0.7 pg/mL), IP-10 (8.6 pg/mL), IL-12p70 (0.6 pg/mL), MIP-1α (2.9 pg/mL), MIP-1β (3.0 pg/mL) and TNFα (0.7 pg/mL). Plates quantifying cytokines in cell culture supernatants used RPMI-1640 media + 10% FBS as the matrix for standard, control and background wells. Data were acquired on a Bio-Plex 200 (Bio-Rad) and analysed with Bioplex Manager software (version 5.0, Bio-Rad). Standard curves for each analyte were generated using 5 parameter logistic regressions. Samples below the limit of detection were assigned a value of half of the detection limit.

### Soluble LAG-3 ELISA

Quantification of sLAG-3 was performed by in-house optimized ELISA. 96 well plates were coated with 5 μg/mL of coating antibody (anti-LAG-3 clone 11E3, Enzo Life sciences) and incubated with 100 μL of sample. A 10-point standard curve was generated using doubling dilutions of recombinant human LAG-3-Fc (Enzo Life Sciences) from 8 ng/mL to 15.6 pg/mL diluted in RPMI-1640 + 10% FBS media. sLAG-3 was detected by the addition of 0.5 μg/mL of anti-LAG-3-biotin (clone 17B4) and streptavidin HRP. Super sensitive TMB (Sigma) was used as the colorimetric substrate and after the addition of a 3% HCl stop solution, the optical density was measured at 450 nm.

### Statistical analysis

Statistical analyses were performed using GraphPad Prism version 6.0. Two and three group comparisons were performed using the non-parametric Mann–Whitney and Kruskal-Wallis (with Dunn’s post-test in the event of a significant Kruskal-Wallis p value) tests, respectively. Correlations were performed using Spearman’s rho value. Comparison of intra-patient (matched) data was carried out by Wilcoxon matched-pairs test (2 groups) or Friedman test (3 groups). Categorical variables (such as the presence or absence of an above-background cytokine response) were compared by chi squared analysis. p values < 0.05 were considered to be statistically significant.

## Additional files

Additional file 1:
**Representative ex vivo staining of PBMC samples to identify lymphocyte populations.** Singlets were identified by FSC-A versus FSC-H plot. Lymphocytes were gated by FSC-A versus SSC-A. The CD3- population was subgated into three NK cell subsets based on CD56 and CD16 expression. The CD3+ population was subgated into CD3 + 6B11+ iNKT cells and CD3 + 6B11- T cells. Both populations were further gated by CD4 and CD8 staining. Additional file 2:
**(A) PD-1 expression on CD8+ T cells among HIV-infected participants compared to healthy controls.** (B) Correlation between PD-1 and LAG-3 expression on CD8+ T cells among all participants. (C) Representative staining of LAG-3 on CD8+ T cells, and PD-1 expression on CD8 + LAG-3+ T cells. (D) PD-1 expression on CD8 + LAG-3+ T cells compared to bulk CD8+ T cells. Statistical comparisons made by Mann–Whitney test, Wilcoxon test and Spearman correlation. *p < 0.05, **p < 0.01, ****p < 0.0001. Additional file 3:
**Representative surface and cytokine staining of iNKT stimulations.** Singlets were identified by FSC-A versus FCS-H gating, followed by gating on the lymphocyte population. Aberrant fluorescence signals were excluded by gating on FITC fluorescence over time. Dead cells were excluded by Live/Dead viability staining. iNKT cells were gated as CD3 + 6B11+. Intracellular staining of IFNγ and TNFα and surface staining of LAG-3 and PD-1 is shown for media (negative control), PMA/ionomycin and αGalCer stimulations. Additional file 4:
**Correlations between** ***ex vivo*** **iNKT LAG-3 and PD-1 expression with 5 day post-αGalCer stimulation cytokine production among all participants.**
Additional file 5:
**Correlation between background-subtracted sLAG-3 concentrations at 5 days post αGalCer-stimulation and supernatant cytokines/chemokines.**
