# Supplementary figures and images for: Elevated expression of LAG-3, but not PD-1, is associated with impaired iNKT cytokine production during chronic HIV-1 infection and treatment
Source: Retrovirology. 2015 Feb 13;12:17. doi: 10.1186/s12977-015-0142-z (PMC4332911; doi:10.1186/s12977-015-0142-z)

Singlets

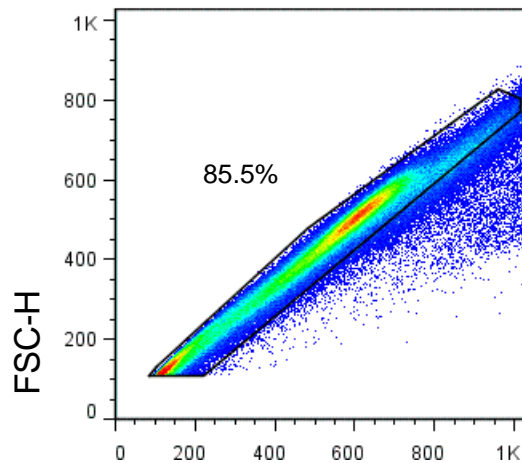

Lymphocytes

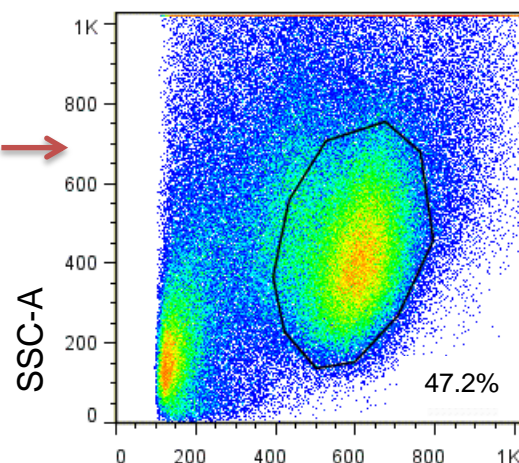

CD3+ and CD3- Subsets

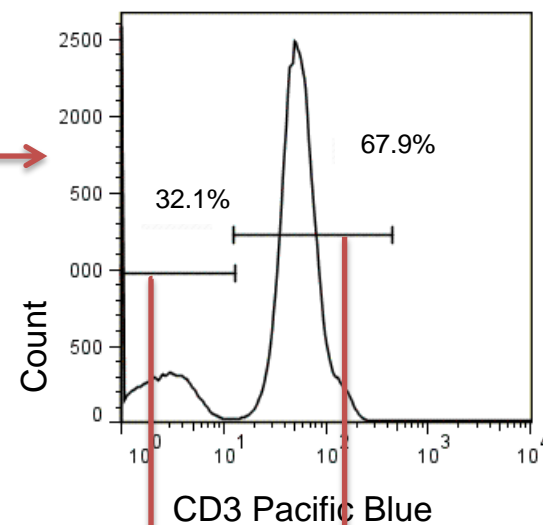

NK Subsets

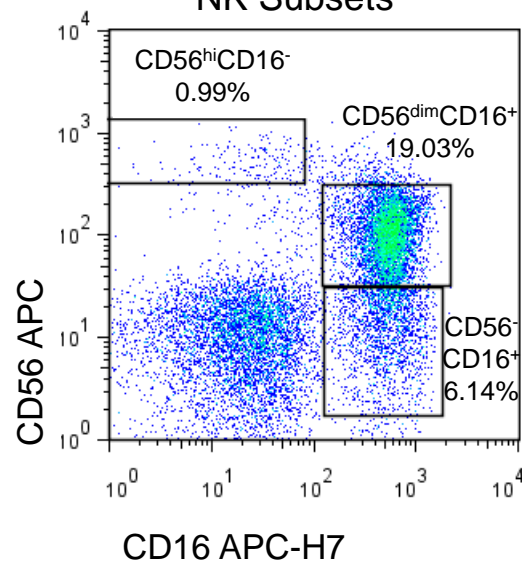

iNKT and T cells

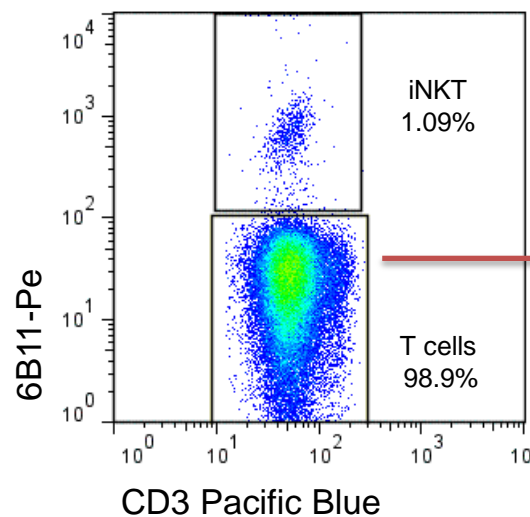

CD4+ and CD8+ T cells

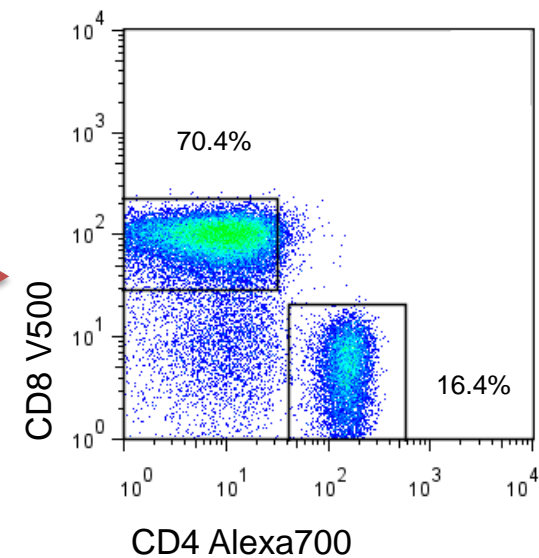

Supplement: Additional file 1: — Representative ex vivo staining of PBMC samples to identify lymphocyte populations. Singlets were identified by FSC-A versus FSC-H plot. Lymphocytes were gated by FSC-A versus SSC-A. The CD3- population was subgated into three NK cell subsets based on CD56 and CD16 expression. The CD3+ population was subgated into CD3 + 6B11+ iNKT cells and CD3 + 6B11- T cells. Both populations were further gated by CD4 and CD8 staining. [file 12977_2015_142_MOESM1_ESM.pdf]

A)

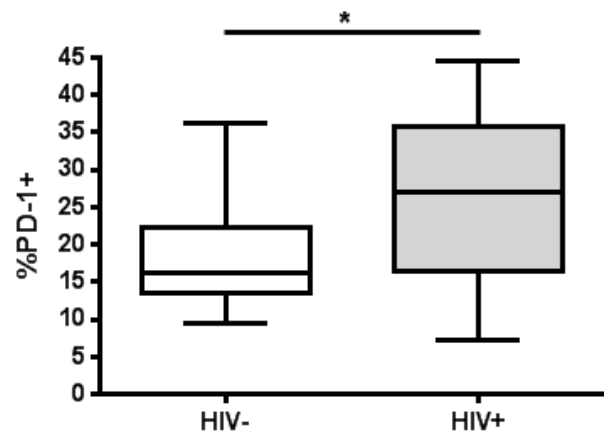

B)

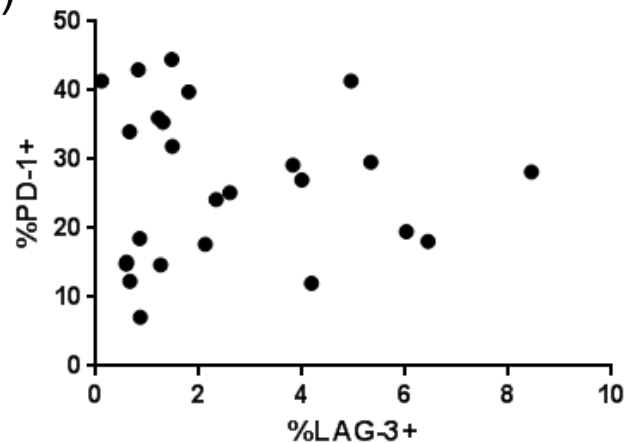

C)

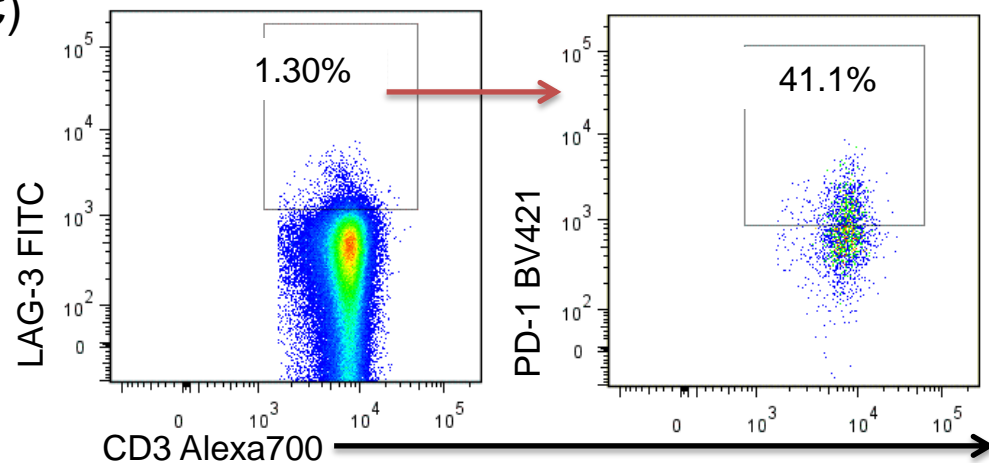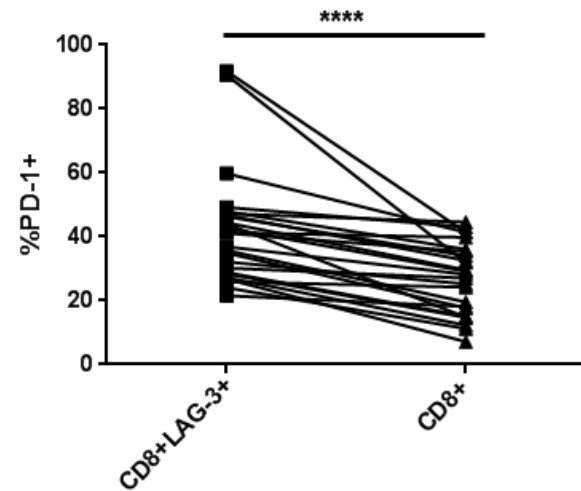

Supplement: Additional file 2: — (A) PD-1 expression on CD8+ T cells among HIV-infected participants compared to healthy controls. (B) Correlation between PD-1 and LAG-3 expression on CD8+ T cells among all participants. (C) Representative staining of LAG-3 on CD8+ T cells, and PD-1 expression on CD8 + LAG-3+ T cells. (D) PD-1 expression on CD8 + LAG-3+ T cells compared to bulk CD8+ T cells. Statistical comparisons made by Mann–Whitney test, Wilcoxon test and Spearman correlation. *p < 0.05, **p < 0.01, ****p < 0.0001. [file 12977_2015_142_MOESM2_ESM.pdf]

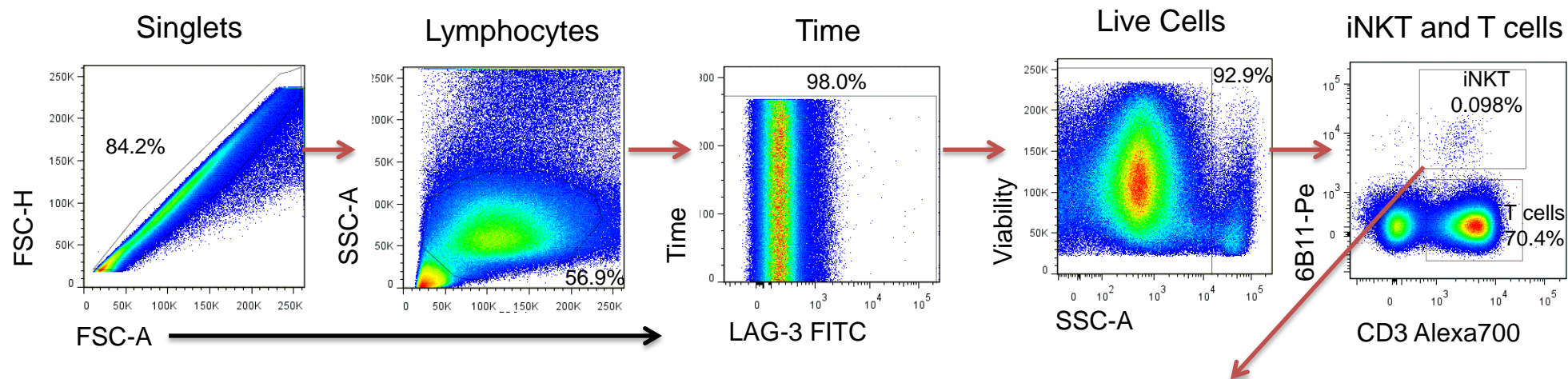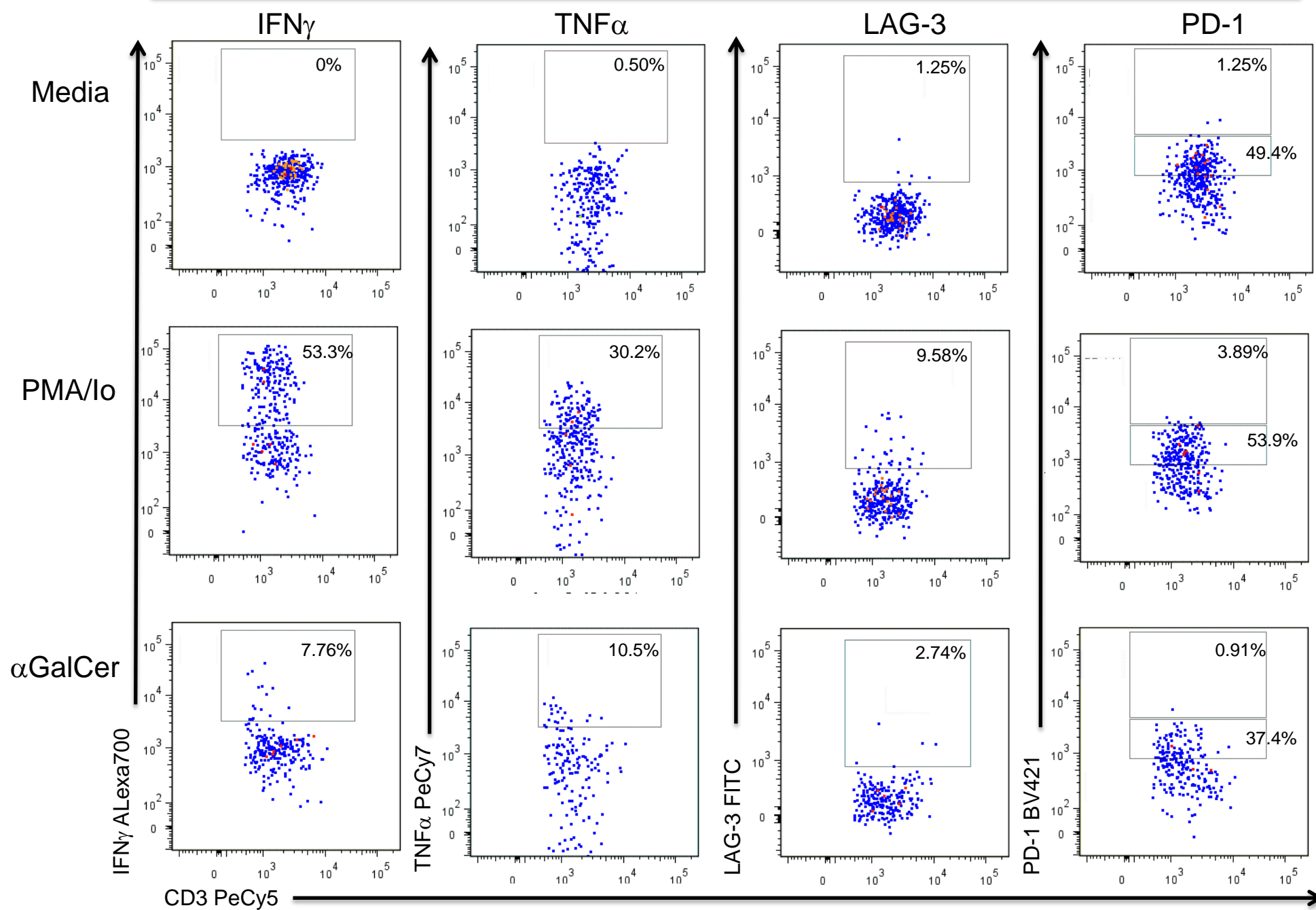

Supplement: Additional file 3: — Representative surface and cytokine staining of iNKT stimulations. Singlets were identified by FSC-A versus FCS-H gating, followed by gating on the lymphocyte population. Aberrant fluorescence signals were excluded by gating on FITC fluorescence over time. Dead cells were excluded by Live/Dead viability staining. iNKT cells were gated as CD3 + 6B11+. Intracellular staining of IFNγ and TNFα and surface staining of LAG-3 and PD-1 is shown for media (negative control), PMA/ionomycin and αGalCer stimulations. [file 12977_2015_142_MOESM3_ESM.pdf]
